# Accelerated detection of *Clostridioides difficile* sequence type 37 by integrating MALDI-TOF mass spectrometry with artificial neural network

**DOI:** 10.1128/spectrum.01728-25

**Published:** 2025-11-13

**Authors:** Liqian Wang, Keqing Zhang, Junjie Lao, Guangzhi Du, Xinghan Huang, Jie Wang, Xianjun Wang, Dazhi Jin, Yu Chen

**Affiliations:** 1 Department of Laboratory Medicine of The First Affiliated Hospital, Zhejiang University School of Medicine 667770, Hangzhou, Zhejiang, China; 2 Department of Laboratory Medicine, Affiliated Hangzhou First People’s Hospital, School of Medicine, Westlake University 681395, Hangzhou, Zhejiang, China; 3 Hangzhou Geriatric Hospital, Department of Laboratory Medicine, Affiliated Hangzhou First People's Hospital Chengbei Campus, School of Medicine, Westlake University 681395, Hangzhou, Zhejiang, China; 4 The Fourth School of Clinical Medicine, Zhejiang Chinese Medical University, Hangzhou First People's Hospital 74630 , Hangzhou, Zhejiang, China; 5 School of Laboratory Medicine, Hangzhou Medical College 680617, Hangzhou, Zhejiang, China; 6 Laboratory Medicine Center, Department of Clinical Laboratory, Zhejiang Provincial People’s Hospital, Hangzhou Medical College 117839, Hangzhou, Zhejiang, China; 7 Key Laboratory of Biomarkers and In Vitro Diagnosis Translation of Zhejiang Province, Hangzhou, Zhejiang, China; 8 Key Laboratory of Clinical In Vitro Diagnostic Techniques of Zhejiang Province, Hangzhou, China; Quest Diagnostics Nichols Institute, Chantilly, Virginia, USA

**Keywords:** *Clostridioides difficile*, multilocus sequence typing, machine learning, artificial neural network

## Abstract

Rapid identification of *Clostridioides difficile* sequence type 37 (ST37), also known as RT017, is crucial due to its association with severe infections and antibiotic resistance. Existing methodologies are labor-intensive and costly. The modeling approach combining matrix-assisted laser desorption ionization time-of-flight mass spectrometry (MALDI-TOF MS) with machine learning offers a promising alternative for fast and cost-effective subtyping. This work gathered 1,155 mass spectra representing 385 distinct clinical *C. difficile* isolates from multiple regions, including 118 ST37 isolates (30.65%) and 267 non-ST37 isolates (69.35%). An artificial neural network (ANN) model was created using MALDI-TOF MS data, trained on 80% of the data set and validated on the remaining 20%. The constructed ANN model demonstrated exceptional diagnostic precision and reliable generalizability, achieving an area under the receiver operating characteristic curve of 0.96 and an area under the precision–recall curve of 0.94 for detecting *C. difficile* ST37 in the validation set. Furthermore, we identified the top 15 potential biomarkers of *C. difficile* ST37 strains with mass-to-charge ratios of 6,729, 12,013, 12,012, 6,731, 7,296, 12,085, 14,716, 7,292, 3,104, 7,293, 16,966, 15,360, 7,259, 18,488, and 14,660 Da. Our model provides a rapid, reliable, and economical option for identifying *C. difficile* ST37. Once species-level identification is completed using MALDI-TOF MS, our ANN model enables rapid subtype classification of ST37 within approximately 10 seconds, significantly reducing overall turnaround time. This facilitates accurate clinical diagnosis, mitigating the risk of severe *C. difficile* infections.

**IMPORTANCE:**

*C. difficile* ST37 (RT017) is a highly virulent strain that often causes severe infections and is frequently resistant to antibiotics such as fluoroquinolones and clindamycin, which are known to promote *C. difficile* infection. Rapid identification of this strain is essential to ensure timely clinical intervention and effective infection control. Current detection methods rely on lengthy and labor-intensive procedures, delaying treatment decisions. This study introduces a new, rapid identification method combining mass spectrometry with machine learning. The developed artificial neural network can accurately distinguish the ST37 strain in approximately 10 seconds, significantly reducing diagnostic time compared to traditional methods. Implementing this fast, reliable, and economical diagnostic tool in clinical laboratories will enhance patient care by facilitating quicker diagnosis and targeted therapy, thus minimizing the risk of severe complications associated with *C. difficile* infections.

## INTRODUCTION

*Clostridioides difficile* infections (CDIs) present a clinical spectrum from mild diarrhea to severe conditions, including toxic megacolon, pseudomembranous colitis, and colonic perforation, requiring timely and precise diagnostic techniques (1). In several developed nations, it has become a leading cause of healthcare-associated infections and diarrhea. For instance, in the United States, the annual hospitalizations and medical costs due to *Clostridioides difficile* are estimated to exceed 200,000 cases and one billion dollars (2). Due to *C. difficile*’s significant resistance to antibiotics, the Centers for Disease Control and Prevention has classified *C. difficile* as an antibiotic-resistant pathogen requiring urgent attention.

Certain sequence types (STs), like sequence type 37 (ST37) (RT017), have been frequently linked to severe CDI cases in clinical settings (3, 4). ST37 strains commonly display resistance to antibiotics like fluoroquinolones, macrolides, and clindamycin (5). Since these agents are not used for treatment, they can promote the selection and proliferation of *C. difficile*, highlighting the need for rapid identification in infection control. Studies suggest that the severity of conditions caused by ST37 is comparable to those caused by the hypervirulent ST1 (ribotype 027) (6). Preliminary research by our team has identified (7) that certain gene variations in the metabolic pathways of the ST37 *C. difficile* strain are associated with severe symptoms of CDI. A 13-country Asia-Pacific survey (600 cases) showed ST37 as the most common isolate (16.7% of isolates) (8). In Southeast Asia, ST37/RT017 is also highly prevalent: Thai studies report ST37 in 30%–42% of toxigenic isolates, and RT017 was the single most frequent strain in an Indonesian study (9). Therefore, the rapid and precise identification of the ST37 strain is important for clinical treatment and prevention of CDI.

Given the propensity of the ST37 strain of *C. difficile* to cause severe infections, it is of paramount importance to rapidly and accurately identify the ST37 strain. Various molecular typing methods, including ribotyping, multilocus sequence typing (MLST), and pulsed-field gel electrophoresis, have been extensively employed for the identification and genotyping of *Clostridioides difficile*. However, these methods are time-consuming, labor-intensive, and costly, which complicates their routine application in clinical settings (10). Consequently, there is a significant need for identification methods that are rapid, simple, and economical. Matrix-assisted laser desorption ionization time-of-flight mass spectrometry (MALDI-TOF MS) offers a rapid, cost-effective, and user-friendly alternative that fits well in clinical microbiology laboratories (11). Given the widespread adoption and excellent performance of MALDI-TOF MS, the primary focus of this technology has shifted from microbial identification toward distinguishing subgroups within the same species, including methicillin-resistant *Staphylococcus aureus* (12), carbapenem-resistant *Klebsiella pneumoniae* (13), *Escherichia coli* (14), and *Pseudomonas aeruginosa* (15). Artificial neural network (ANN) models stand out in medical research for their superior accuracy, architectural flexibility, and the ability to be fine-tuned with techniques like early stopping, weight decay, and dropout layers, which collectively contribute to reduced overfitting and improved generalization capabilities (16). Applying MALDI-TOF MS with ANN in bloodstream infections reduced 30-day mortality, hospital stay, and infection-related costs (17).

Recent studies have focused on the rapid identification of *C. difficile* subtypes using MALDI-TOF MS, including efforts to classify hypervirulent ribotypes such as RT027 and RT176 and to distinguish RT017 using ClinProTools (18–21). Calderaro et al. and Zautner et al. also demonstrated the feasibility of subtype-level differentiation using MALDI-TOF MS in a clinical setting, highlighting the growing interest in this domain (22, 23). However, limitations including small sample sizes, regional confinement, restricted genetic diversity, lack of external validation, and the failure to employ artificial neural networks to mitigate overfitting and improve generalizability have adversely affected the quality of these analyses. This study involved the collection of strains from multiple regions, encompassing various common ST strains and reference strains, along with external validation. Furthermore, by employing an artificial neural network model, we have improved its performance and generalizability in the real world.

This project seeks to tackle the aforementioned challenges by developing a new classification scheme to differentiate *C. difficile* ST37 from non-ST37 types, utilizing the integration of MALDI-TOF MS and machine learning (ML) with ANN models. In our new scheme, after species confirmation with MALDI-TOF MS, the ANN model processes the spectral data and classifies isolates as ST37 or non-ST37 within approximately 10 seconds. The rapid identification process aids physicians in the timely and effective diagnosis and treatment of CDI attributed to the ST37 strain, consequently minimizing the risk of severe *C. difficile* infections.

## RESULTS

### Collection of clinical *C. difficile* isolates

This study analyzed 385 clinical isolates of *C. difficile* collected by our team over 8 years. Among these, 118 isolates (30.65%) were identified as the ST37 (RT017), while 267 isolates (69.35%) belonged to non-ST37 types. The non-ST37 isolates comprised 18 commonly encountered clinical subtypes, with ST3 (52 isolates) and ST54 (45 isolates) being the most frequent. Of the total isolates, 362 were sourced from various hospitals in Zhejiang Province, and 23 originated from other regions. Detailed information is presented in Table 1.

**TABLE 1:** Sources and MLST sequence types of clinical isolates of *C. difficile* in this study

Sample source	MLST type	Number of strains
China, Zhejiang Province	ST37	95
	Non-ST37	267
Hebei Province	ST37	12
Hunan Province	ST37	4
Hong Kong	ST37	2
South Korea	ST37	3
Singapore	ST37	1
United States	ST37	1

### Establishment and performance analysis of the ANN model for *C. difficile* ST37 and non-ST37 strains

In the traditional microbiological workflow, identifying *C. difficile* ST37 typically requires 5–6 days. This process includes bacterial culture, identification using MALDI-TOF, isolation and subsequent reculturing of the strain, DNA extraction, PCR amplification, and sequencing analysis. Our ANN model can expedite this process by at least 3 days compared to the conventional approach. As such, this model holds promise for aiding in the screening of *C. difficile* ST37 and assisting clinicians in promptly addressing the risk of severe infections caused by this strain.

**Fig 1:**
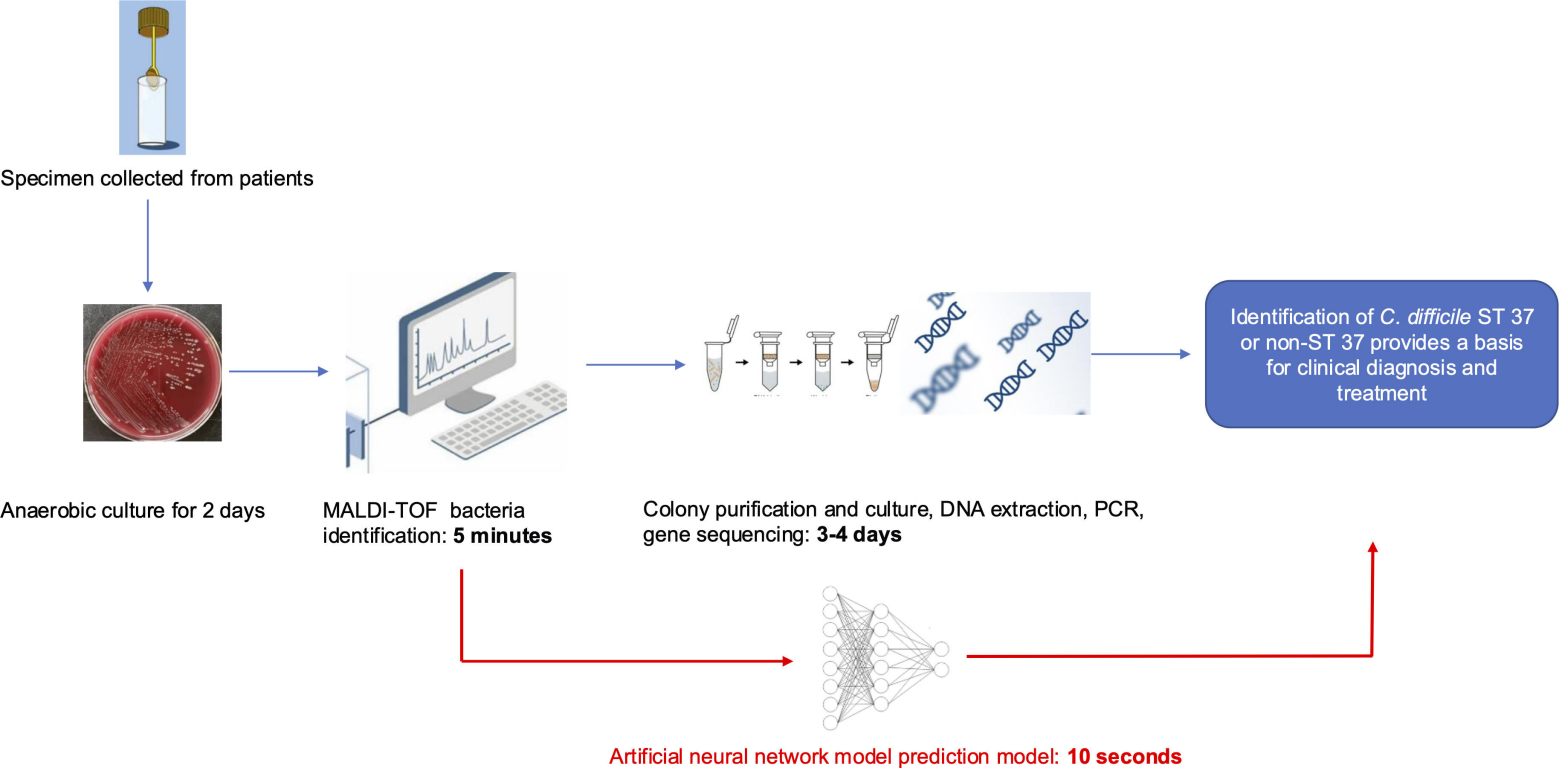
Illustration of the existing workflow in clinical laboratories at Hangzhou First People’s Hospital, where specimens are collected from the bedside to generate an AST report, a process that typically requires a minimum of 5 days. Then, clinicians can determine whether vancomycin treatment is needed to prevent the occurrence of severe *C. difficile* infection symptoms. Integrating our model into this workflow in the red frame section, we can screen for *C. difficile* ST37 isolates before the PCR results and guide clinicians.

In this study, we propose a novel approach for detecting *C. difficile* ST37 isolates (Fig. 1). Figure 1 illustrates the current workflow in the clinical laboratory of Hangzhou First People’s Hospital, where the identification process from bedside specimen collection to final identification takes at least 5–6 days. By incorporating our neural network model into this workflow, the new approach can reduce the time required for detecting *Clostridioides difficile* ST37, providing clinicians with critical diagnostic and treatment information, thereby reducing the risk of severe CDI.

We utilized 80% of the total data set (*n* = 921), consisting of 282 ST37 mass spectra and 639 non-ST37 mass spectra, to train an ANN model capable of efficiently differentiating between *Clostridioides difficile* ST37 and non-ST37. The ANN model achieved a sensitivity of 0.99, specificity of 0.96, accuracy of 0.97, F1 score of 0.95, and area under the receiver operating characteristic curve (AUROC) of 0.99, indicating its high reliability in differentiating *C. difficile* ST37 from other subtypes.

**Fig 2:**
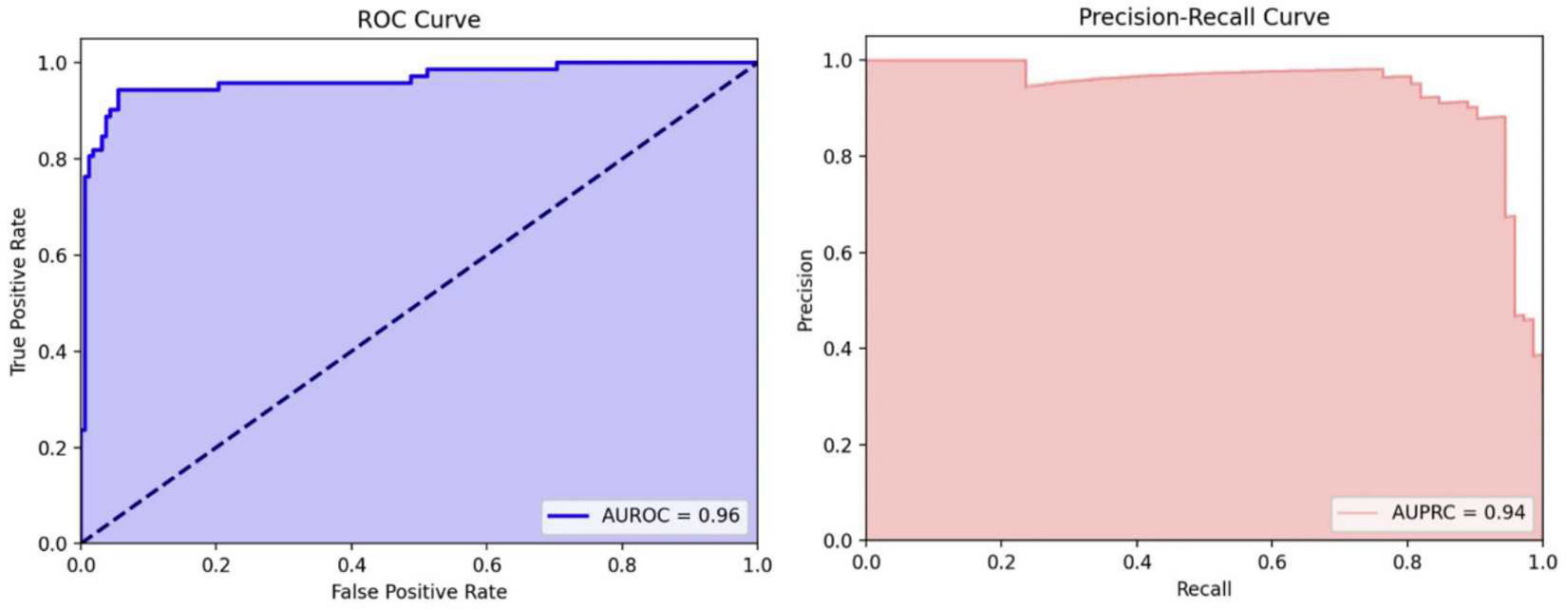
(Left) The receiver operating characteristic curve (ROC) area, denoted as AUROC, indicates the effectiveness of the ANN model in distinguishing *C. difficile* ST37 isolates. During external validation, the AUROC achieved was 0.96. (Right) The area under the precision–recall curve (AUPRC) is utilized to evaluate machine learning models, particularly when dealing with imbalanced data sets. The AUPRC, measured at 0.94 during the external validation phase, demonstrates the model’s robustness even in the context of data set imbalance.

We used the remaining 20% of the data (*n* = 234), consisting of 72 ST37 mass spectra and 162 non-ST37 mass spectra, as an independent validation data set for external validation. Our neural network model achieved a sensitivity of 0.94, specificity of 0.94, accuracy of 0.94, F1 score of 0.91, AUROC of 0.96 (Fig. 2, left), and area under the precision–recall curve (AUPRC) of 0.94 (Fig. 2, right).

### Analysis of significant peaks

**Fig 3:**
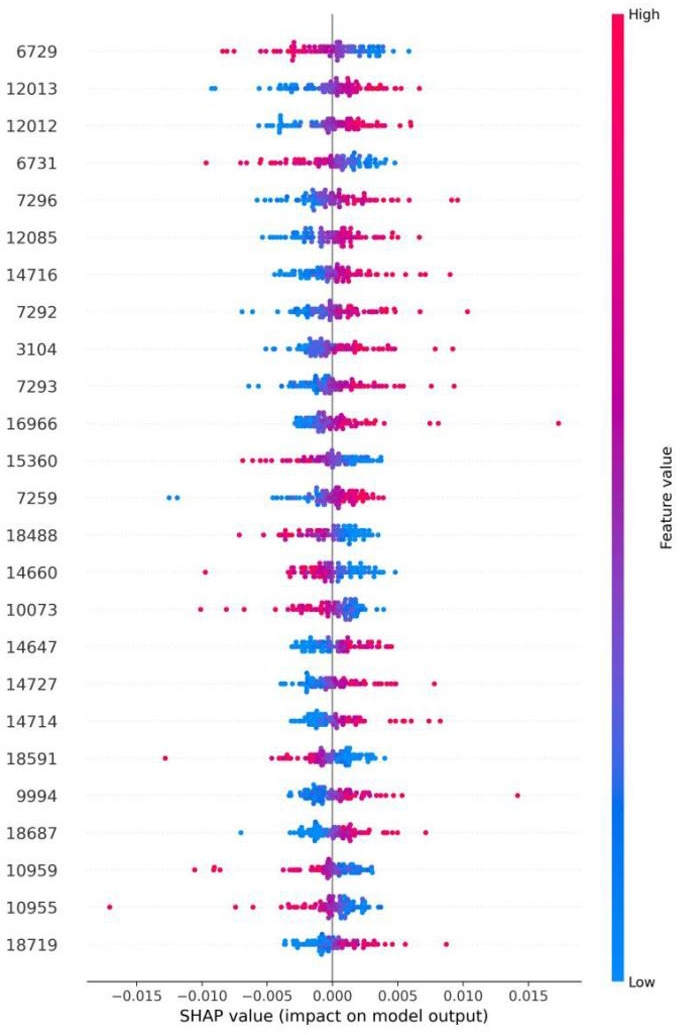
Shap values, an explainable AI technique, are utilized to assess the influence of individual features in models, enhancing the transparency and interpretability of machine learning systems. The provided illustration shows the Shap values for the top 25 features in our ANN model, which could offer significant insights for future research.

Further analysis of the ANN model was performed using the Shap package in Python v.3.7 to identify potential biomarkers and assess the significance of specific peaks. As shown in Fig. 3, isolates exhibiting characteristic mass spectral peaks are highlighted in red, whereas those lacking such features are indicated in blue. To improve model interpretability and transparency, Shap values were utilized to quantify the contribution of individual spectral peaks. Figure 3 presents the Shap values for the top 25 features of the ANN model, offering potential insights for future research. The model’s predictive performance for identifying *C. difficile* ST37 improves as the distance from the Shap value zero point increases. In this study, the 25 most promising biomarker candidates were pinpointed within clinical isolates of the ST37 strain of *C. difficile*, which are 6,729, 12,013, 12,012, 6,731, 7,296, 12,085, 14,716, 7,292, 3,104, 7,293, 16,966, 15,360, 7,259, 18,488, 14,660, 10,073, 14,647, 14,727, 14,714, 18,591, 9,994, 18,687, 10,959, 10,955, and 18,719 Da.

## DISCUSSION

The highly virulent *C. difficile* ST37 (RT017) strain is prone to causing severe *C. difficile* infections. However, current testing methods for the ST37 strain are time-consuming, which delays timely clinical treatment. In this research, we have successfully developed a model that is both rapid and precise, as well as cost-effective. This model was created by applying ML to analyze mass spectrometry peak data from 1,155 samples, which were collected from 385 strains of the bacteria isolated in various regions. The purpose of the model is to identify the hypervirulent strain ST37 of *C. difficile*. By deploying this model, the diagnostic time for CDI caused by the *C. difficile* ST37 strain has been significantly reduced from the typical 3–4 days required by gene sequencing analysis to a mere 10 seconds. This advancement contributes significantly to outbreak tracking and epidemiological surveillance.

MALDI-TOF MS combined with machine learning has been proven effective for bacterial subtype identification, as demonstrated in studies on methicillin-resistant *Staphylococcus aureus* (24–26) and carbapenem-resistant *Klebsiella pneumoniae* (13), underscoring its potential in clinical microbiology. These studies offer significant support for clinical decision-making, early intervention, and the enhanced management of infectious diseases. However, the use of MALDI-TOF models for classifying *C. difficile* subtypes has been remarkably infrequent. The only study conducted on this, published in September 2023 (18), primarily classified European hypervirulent strains RT027/RT176, RT023, and RT045/078/126/127 but did not encompass the ST37 (RT017) strain, which is more common in Asia. Consequently, our research, leveraging MALDI-TOF MS techniques in conjunction with ML approaches, focuses on the identification of the *C. difficile* ST37 strain, thereby providing a crucial foundation for the rapid differentiation of clinical microbiological specimens.

ST37 is among the most prevalent sequence types of *C. difficile* identified in Asia, and it exhibits a resistance profile that is notably distinct from that of other sequence types. The swift identification of *C. difficile* ST37 is of paramount importance in clinical settings (5, 27). Compared to earlier studies such as that of Li et al., which applied MALDI-TOF MS with ClinProTools for subtype-level classification, our study introduces several advancements (19). Notably, we employed an ANN model that achieved high diagnostic performance (sensitivity = 0.99, specificity = 0.96 in the training set) and validated the model using an external data set. In addition, the use of a large and geographically diverse collection of 385 clinical *C. difficile* isolates with 1,155 spectra significantly enhances model generalizability. These provide a more robust and scalable solution for subtype-level identification. Prior studies such as that of Zautner et al. primarily focused on European isolates, including ribotypes RT027 and RT176, but did not evaluate RT017, likely due to its low prevalence in most European cohorts (23). In contrast, ST37 (RT017) is among the most frequently isolated strains in many Asia-Pacific regions, underscoring the regional relevance of our study. Other epidemiologically important sequence types such as ST1 (RT027) merit inclusion; however, the underrepresentation in our isolate collection limited our ability to construct a robust multiclass model. Future work will aim to expand the data set to enable broader subtype classification.

In contrast to models like decision trees, random forests, and support vector machines, artificial neural networks demonstrate a stronger capacity for optimization, enabling them to more effectively prevent overfitting and promote broader model generalization (28). Although MALDI-TOF MS offers an efficient and convenient approach for the identification of microbial subtypes, technical variations can occur throughout the analysis process, from sample preparation to the instrument conditions used with the equipment, due to the imprecision of different steps and conditions (29). To reduce these technical discrepancies, computational procedures, such as normalization of the obtained spectra, are commonly employed. Machine learning models, particularly those with the flexible architecture of ANNs, offer precise methodological support for data analysis with MALDI-TOF MS. It is also noteworthy that the lack of an independent validation data set may result in overfitting the training data set, particularly when the data set size is small, potentially limiting the model’s performance and generalizability in practical applications. The lack of external validation is a commonly addressed issue in many studies. As an illustration, a systematic review released in October 2020 reported that merely about 11% (4 out of 36) of machine learning applications involving MALDI-TOF MS underwent external validation (30). To assess the practical utility of our model, we conducted external validation by testing it on a separate, independent data set. Furthermore, we employed an artificial neural network analysis model, enhancing the robustness of our methodology in comparison to previous studies (12).

In this study, the potential biomarkers identified for *C. difficile* ST37 isolates differ from those reported by Li et al. (19). Factors contributing to these discrepancies may include the genetic diversity among bacterial strains from different regions and the limited generalizability of models used in previous research. Moreover, our modeling approach differs from that of Li et al. They employed ClinProTools software for model construction, whereas our study utilized an ANN model. Compared to ClinProTools, the ANN model is more adept at uncovering nonlinear relationships between inputs and outputs, which is particularly effective for complex mass spectrometry data analysis. Additionally, the ANN possesses a more robust learning capacity and, through data preprocessing and meticulous parameter tuning, enhances the predictive performance of the model. We selected an ANN instead of a convolutional neural network (CNN) because our input data consisted of one-dimensional spectral vectors, rather than two-dimensional spatial structures typical of image data. While CNNs are highly effective for spatially organized inputs, ANN has been shown to perform well on one-dimensional data such as MALDI-TOF MS spectra (31).

A review analyzed 36 studies published before 31 January 2020, which focused on the integration of machine learning with MALDI-TOF MS data. The review highlighted several common limitations within these studies, such as insufficient sample size, incomplete coverage of pathogen genetic diversity, and a lack of external validation (30). In contrast, this study aimed to address these issues by collecting bacterial strain samples from various regions to enhance genetic diversity and by establishing independent external validation to prevent model overfitting, thereby improving the model’s generalizability and robustness. Fecal PCR assays can significantly shorten the time for *C. difficile* detection to a few hours; however, our method offers several important advantages. First, while fecal PCR is excellent for rapid toxin gene detection, it is generally not designed for high-resolution strain typing, such as identifying ST37 (32). Our MALDI-TOF MS-based approach, combined with neural networks, enables direct identification of the ST37 strain from cultured colonies. This is essential for tracking hypervirulent lineages in clinical settings. Second, our method is more cost-effective in many laboratories. MALDI-TOF systems are already in routine use for species identification. Our model builds on this platform without the need for extra reagents or equipment. In contrast, PCR requires specific primers, probes, and extraction kits, increasing cost and complexity. Third, our method preserves bacterial isolates for downstream testing. This includes antimicrobial susceptibility testing and genomic analysis, which PCR-based methods cannot provide. Therefore, while PCR is faster for species-level detection, our approach offers clear advantages in accuracy, cost, and clinical utility for ST37 surveillance.

While our approach shows promise for rapid and accurate detection of *C. difficile* ST37, several limitations should be acknowledged. First, the number of bacterial strains collected remains limited, and the data set is geographically limited. Future efforts should aim to obtain more strain data from a wider range of hospitals, regions, and countries to train the machine learning models and enhance their generalization capabilities. Second, our model was developed using spectra from a single MALDI-TOF platform, which could impact cross-platform reproducibility, and environmental variables such as culture conditions could influence protein profiles. Third, the “black-box” nature of the ANN may limit interpretability in some clinical settings, although Shap values have been done. Lastly, the study used retrospective data; prospective, real-world validation remains essential before clinical deployment.

In summary, the artificial neural network model we developed, which combines MALDI-TOF MS technology with MLST sequencing data, performed exceptionally well in accurately identifying *C. difficile* ST37. The model achieved perfect recognition results in the training set, with both AUROC and AUPRC reaching 0.999; in the validation set, the model demonstrated good discriminative power with an AUROC of 0.96 and an AUPRC of 0.94. This model enables rapid preliminary screening for *C. difficile* ST37 within 10 seconds, offering a significant advantage over conventional methods that require 3–4 days, thereby enhancing timely clinical decision-making and patient care. This simple, fast, and cost-effective method significantly enhances the clinical accuracy of diagnosis and treatment of *C. difficile* ST37 infections and supports public health departments in better disease prevention and control.

## MATERIALS AND METHODS

### Data source

Our research group selected 385 previously isolated *C. difficile* strains. Of these, 162 strains (42.08%) were from Hangzhou First People’s Hospital; 200 strains (51.95%) were from various other hospitals in Zhejiang Province; 12 strains were from Hebei Province; 4 strains were from Hunan Province; 2 strains were from Hong Kong, China; 3 strains were from South Korea; 1 strain was from Singapore; and 1 strain was from the United States. This ensures a diverse representation for robust model training and validation. Quality control strains included ATCC BAA-1870, 9689, 43598, 43255, BAA-1812, BAA-1382, and BAA-1804. Twenty-three strains from outside Zhejiang Province and seven quality control strains were kindly provided by Dr. Dazhi Jin from the School of Laboratory Medicine, Hangzhou Medical College.

### Detection of *C. difficile* multilocus sequence typing

MLST was employed in this study. Control strains of *Clostridioides difficile* (ATCC BAA-1870, 9689, 43598, 43255, BAA-1812, BAA-1382, and BAA-1804) served as references. A 30 µL aliquot of the preserved *C. difficile* solution was transferred onto a blood agar plate using a pipette. The sample was streaked across four zones and incubated under anaerobic conditions at 37°C for 48 hours to obtain isolated single colonies. For MLST, seven loci (adk, atpA, dxr, glyA, recA, sodA, and tpi) were amplified via PCR as described previously (33). The allele and ST data for *C. difficile* were then deposited in a publicly accessible MLST database (http://pubmlst.org/cdifficile).

### Identification of *C. difficile* through MALDI-TOF MS

Using a pipette, 30 µL of the preserved *C. difficile* solution was added to a blood agar plate. The sample was streaked in four zones and incubated anaerobically at 37°C for 48 hours to isolate single colonies. This process was repeated once. Then, the samples were prepared using the formic acid extraction method. In brief, three single colonies with an inoculating loop were suspended in 300 µL of ultrapure water in an EP tube, pipetted up and down repeatedly, and vortexed for at least 1 minute to form a uniform suspension. Then, 900 µL of anhydrous ethanol was added; the mixture was vortexed for at least 1 minute to mix well, and the microbial extract was centrifuged at 13,000 rpm for 2 minutes to remove the supernatant. The centrifugation was repeated to completely remove the ethanol solution, then air-dried in a biosafety cabinet for 30 minutes. Fifty microliters of 70% formic acid was added to the pellet, pipetted up and down to mix, and after vortexing for 1 minute, 50 µL of acetonitrile was added and vortexed for 1 minute to mix thoroughly. The supernatant containing the bacterial protein extract was transferred to another EP tube. One microliter of the bacterial protein extract was added to the MALDI target plate and air-dried at room temperature. One microliter of IVD HCCA matrix solution was added to each sample well and air-dried at room temperature. MALDI-TOF MS was conducted using the MALDI-TOF mass spectrometer (Microflex LT; Bruker Daltonik, Germany). The spectra were processed with MALDI Biotyper Compass software v.4.1. For MALDI-TOF MS analysis, we adhered to the manufacturer’s recommended settings. Each mass spectrum was composed of 240 laser shots, captured in 40-shot increments (linear positive mode, accelerating voltage at +20 kV, and nitrogen laser frequency at 60 Hz). Calibration of the MALDI-TOF MS was conducted using the bacterial test standard before analyzing clinical samples to ensure accurate identification of *C. difficile*. Following the manufacturer’s guidelines, only isolates with identification scores exceeding 2 as *C. difficile* were included as analytes in our study. Following the described procedure, three mass spectra were obtained from distinct positions of each bacterial strain.

### MALDI-TOF MS data preprocessing

The raw mass spectrometry data were initially preprocessed using the R packages MaldiQuant v.1.21 and MALDI quant Foreign v.0.13 (34). This involved a square root transformation of the raw intensity data, smoothing with a Savitzky-Golay filter, and baseline correction using a sensitive nonlinear iterative peak-clipping algorithm. Following the R-based preprocessing, additional data preprocessing and model construction were performed in Python v.3.11. Initially, spectra were trimmed to the range of 2,000–20,000 Da, and zero values were assigned to missing intensity data to avoid errors in the ANN. Averaging the spectral intensity could result in the loss of information, which might reduce our model’s effectiveness. Therefore, for each isolate’s mass spectrometry data, each 1 Da interval was treated as a feature, resulting in 18,000 vectors spanning from 2,000 to 20,000 Da.

The StandardScaler from the Python Scikit-Learn package was then used to convert the intensities into *Z*-scores for each feature. Subsequently, the MALDI-TOF spectra were classified into two groups based on the MLST results (ST37 and non-ST37), and a minimum intensity threshold was set to identify meaningful peaks that could distinguish between ST37 and non-ST37 isolates. We then calculated the proportion of meaningful peaks for each vector within the ST37 and non-ST37 groups to enhance the ML model’s performance. Using the interquartile range (IQR), each vector was categorized into different groups (Q1, Q2, Q3, and Q4) based on the proportion of meaningful peaks in the two groups (34). Our optimal model performance was achieved using vectors scoring above Q3 (>75%, 4,500/18,000). Finally, these 4,500 vectors were utilized as features to construct our ML model.

### Construction of the ANN model

Using Python v.3.11, we developed an ANN model with strains randomly partitioned (random state  =  20) into 80% training and 20% validation sets. The three mass spectrometry spectra of the same strain were placed in the same data set, stratified by ST37 and non-ST37 labels to ensure balance between the data sets. The independent validation data set was not utilized during model training but was reserved exclusively for external evaluation of the model’s performance. An ANN architecture was implemented for ST37 prediction using 4,500 features selected via IQR. The network included an input layer, two hidden ReLU layers, and a softmax output for binary classification (ST37 vs non-ST37).

### Model evaluation

To enhance model robustness and mitigate feature loss, 10-fold cross-validation was applied during training. Post-training, external validation was performed on a randomly shuffled test set (random state  =  1). The model’s effectiveness was evaluated through metrics such as sensitivity, specificity, accuracy, F1 score, the AUROC, and the AUPRC.

### Statistical analysis

The machine learning model’s performance was assessed using the receiver operating characteristic curve, while the precision–recall curve was employed to evaluate the model’s performance under conditions of imbalanced data distribution. The effectiveness of our predictive model was determined using metrics such as sensitivity, specificity, and accuracy, defined in Table S1.

## SUPPLEMENTAL MATERIAL

Table S1 spectrum.01728-25-s0001.docx Performance metrics and their definitions.

ASM does not own the copyrights to Supplemental Material that may be linked to, or accessed through, an article. The authors have granted ASM a non-exclusive, world-wide license to publish the Supplemental Material files. Please contact the corresponding author directly for reuse.
