# Supplementary material for: Accelerated detection of Clostridioides difficile sequence type 37 by integrating MALDI-TOF mass spectrometry with artificial neural network
Source: Microbiol Spectr. 2025 Nov 13;14(1):e01728-25. doi: 10.1128/spectrum.01728-25 (PMC12772325; doi:10.1128/spectrum.01728-25)
Supplement: Table S1 — Performance metrics and their definitions. [file spectrum.01728-25-s0001.docx]

Supplementary Table 1. Performance Metrics and Their Definitions

| Metric | Definition |
| --- | --- |
| Sensitivity | True Positives (ST37) / [True Positives (ST37) + False Negatives] |
| Specificity | True Negatives (non-ST37) / [True Negatives (non-ST37) + False Positives] |
| Accuracy | True Positives (ST37) + True Negatives (non-ST37)] / [True Positives (ST37) + True Negatives (non-ST37) + False Positives + False Negatives] |
